# The Effect of the Extraction Temperature on the Colligative, Hydrodynamic and Rheological Properties of Psyllium Husk Mucilage Raw Solutions

**DOI:** 10.3390/molecules30153219

**Published:** 2025-07-31

**Authors:** Anna Ptaszek, Marta Liszka-Skoczylas, Urszula Goik

**Affiliations:** 1 Centre for Innovation and Research on Prohealthy and Safe Food, University of Agriculture in Krakow, Balicka 104, 30-149 Krakow, Poland; 2 Department of Engineering and Machinery in Food Industry, Faculty of Food Technology, University of Agriculture in Krakow, Balicka 122, 30-149 Krakow, Poland; marta.liszka-skoczylas@urk.edu.pl (M.L.-S.); urszula.goik@urk.edu.pl (U.G.)

**Keywords:** mucilage, *Plantago ovata* husk, extraction temperature, average molecular mass, virial coefficients, rheological time constants, normal force

## Abstract

The aim of the research was to analyse the effect of different extraction temperatures on the colligative, hydrodynamic, and rheological properties of a water-soluble AXs fractions. The research material consisted of raw water extracts of arabinoxylans obtained from the husk at the following temperatures: 40 °C (AX40), 60 °C (AX60), 80 °C (AX80), and 100 °C (AX100). These were characterised in terms of their hydrodynamic, osmotic, and rheological properties, as well as the average molecular mass of the polysaccharide fractions. An increase in extraction temperature resulted in an increase in weight-average molecular mass, from 2190 kDa (AX40) to 3320 kDa (AX100). The values of the osmotic average molecular mass were higher than those obtained from GPC, and decreased with increasing extraction temperature. The dominance of biopolymer–biopolymer interactions was evident in the shape of the autocorrelation function, which did not disappear as the extraction temperature and concentration increased. Furthermore, the values of the second virial coefficient were negative, which is indicative of the tendency of biopolymer chains to aggregate. The rheological properties of the extracts changed from being described by a power-law model (AX40 and AX60) to being described by the full non-linear De Kee model (AX80 and AX100).

## 1. Introduction

The genus Plantago contains more than 200 plant species, and the seeds and husk of the plantain (*Plantago ovata*) are used as a functional ingredient in food products [1,2,3] as well as in the cosmetic and pharmaceutical industries [4,5,6]. The main component of Plantago husks are arabinoxylans (AXs), which, when hydrated, form dietary fibre (mucilage) [2,7]. Seventy percent of the mucilage are AXs loosely bound to the cell wall and therefore water soluble. Other AXs may be covalently and hydrogen bonded to other cell wall components (proteins, lignins, and lignans) and remain insoluble in cold or hot water [5,8].

The polysaccharide that forms the mucilage is a polysaccharide consisting mainly of (1⟶4) linked β-D-xylose, with side chains at positions C-3 or C-2, containing arabinose (A) and xylose (X) in varying proportions [9,10]. AX chains can be substituted by other sugars, such as glucose, mannose, galactose, etc., and can also have acetyl groups and uronic acids, such as glucuronic acid [5,7,9,10]. In the extraction of arabinoxylans and polysaccharides from plant material, ultrasounds, microwaves, or pulsed electric field can be used [11,12,13,14,15], subcritical conditions [16], enzymes [17,18,19] as well as alkaline conditions [17,18,20]. One of the most commonly used methods is hot water extraction (HWE) [20,21,22,23,24]. The molecular properties of the mucilage depend on the botanical origin of the raw material and extraction conditions: the type of raw material (husk or seed) [4,21,22,24,25,26], solvent and raw material/solvent ratio [21,23,24], and temperature [10,23]. Van-Craeyveld et al. [20] reported that the extraction yield was affected by husk suspension concentration and pH rather than by temperature. Enzyme-assisted extraction using various hydrolases allows for obtaining fractions with a lower molecular weight, and the extraction conditions (temperature and pH) are milder than in typical chemical methods [20]. The extraction conditions determine the average molecular mass $M_{W}$ [27] and the arabinose/xylose (A/X) ratio [10,21,24], which increases with the the extraction temperature [21]. Due to its high water absorption capacity and its ability to form gels [9,10], *Platnago ovata* AXs can be used as a multifunctional food additive to modify the mechanical properties of food and stabilise multiphase food systems [1].

The selected functional properties of *Plantago ovata* mucilage solutions have been studied using a variety of measurement techniques, including capillary viscometry and rotational rheometry [10,22,23,28,29], static light scattering (SLS) [10], dynamic light scattering (DLS) [22,29], small angle X-ray scattering (SAXS) [30], and membrane osmometry [29]. According to the results of the study, the conformation that the chains adopt in the solvent and their concentration play a decisive role in shaping the properties of mucilage solutions [10,21,29,30]. The biopolymer–solvent and biopolymer–biopolymer interactions are dependent on the molecular structure—the length of the main (linear) chain and the presence and nature of branching [4,22,25,31]. The second factor is the concentration of the biopolymer, which, together with the affinity of the solvent (good/bad solvent), determines the (bio)polymer–polymer interactions. Due to the nature of the interactions in the physicochemistry of (bio)polymers, three concentration ranges are distinguished—dilute, semidilute, and concentrated—separated by two critical concentrations (c* and c**). For the water-soluble mucilage extracted from *Plantago ovata* seeds and husk, the first critical concentration (c*) was determined, the value of which depended on the extraction temperature [10,29,30]. The rheological properties of mucilage solutions in these three concentration ranges can vary from those characteristic of Newtonian and simple shear thinning fluids (dilute range), rheologically unstable shear thinning (semi dilute) [29,30], to viscoelastic or viscoelastic-elastic (concentrated range) [10,21,30]. To the best of the authors’ knowledge, there is no information in the literature on the effect of extraction temperature on the weight average molecular mass of AXs and the colligative and rheological properties of raw AXs extracts. Knowledge of the impact of the extraction temperature of AXs on the ability of the extract to bind (absorb) water, as well as its rheological properties, particularly apparent viscosity, is crucial when designing new food products. Eliminating E-number thickeners by introducing new fibre-like substances requires investigating interactions in aqueous solutions and subsequently with other food additives. Rheological properties are essential for optimising mechanical operation parameters, including mixing and transport (flow through pipes).

The aim of the research was to analyse the effect of different extraction temperatures on the colligative, hydrodynamic, and rheological properties of a water-soluble AXs fractions. The material studied were the raw aqueous extracts obtained at four selected temperatures: 40 °C, 60 °C, 80 °C, and 100 °C. The starting point for the phenomenological interpretation were the changes of the weight and osmotic average molecular masses determined for the polysaccharide fractions as a function of the extraction temperature. The values of the diffusion coefficients were estimated from the Kohlrausch–Williams–Watts model. The normal forces and apparent viscosity of the AXs fractions were measured and the changes of apparent viscosity were analysed with the help of the temperature scaling method and the De Kee model.

## 2. Results

**Figure A1:**
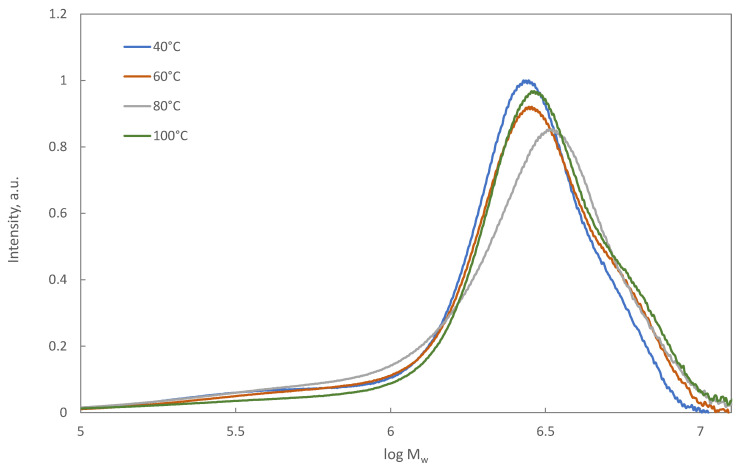
The molecular masses distributions for raw extracts.

The molecular characteristic of arabinoxylans extracted at different temperatures included the determination of the weight ($M_{w}$) and number ($M_{n}$) average molecular masses by GPC. The results are given in Table 1. With increasing extraction temperature, the weight average molecular mass increased from 2190 kDa at 40 °C to 3320 kDa at 100 °C. No such trend was found for the number average molecular mass $M_{n}$. The extract obtained at 100 °C (AX100) had the highest $M_{n}$ value with the lowest dispersity value. In contrast, AX80 with the highest dispersity had the lowest value $M_{n}$. The molecular masses distribution curves are shown on the figure in Appendix A.2 (Figure A1). The profiles of the extracted polysaccharides were single-modal, with the global maximum depending on the extraction temperature and ranging from 1000 kDa to 10,000 kDa. The shape of the distribution function indicated the presence of a fraction characterised by lower molecular masses (170–1000 kDa).

**Figure 1:**
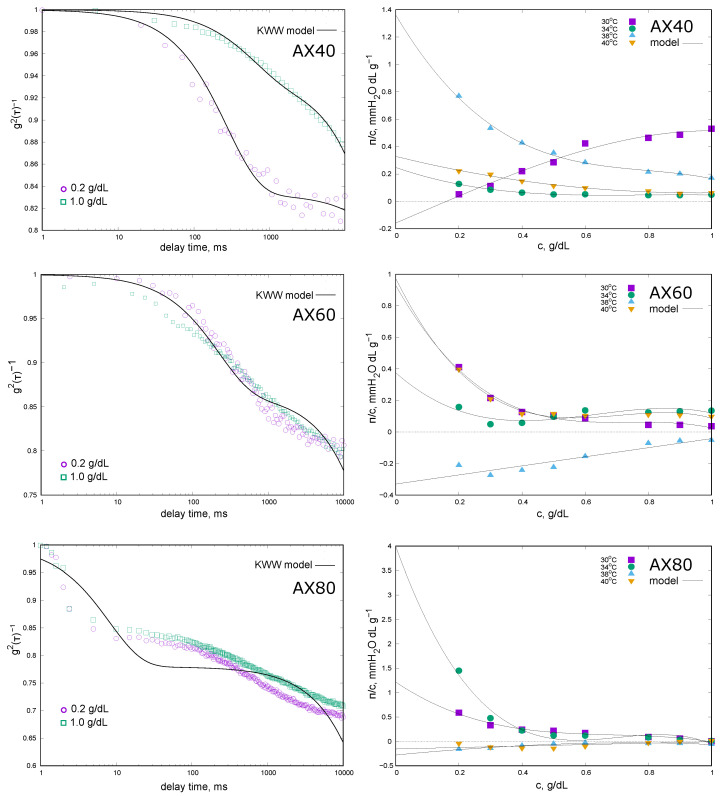
Hydrodynamic and osmotic properties of raw extracts obtained at different temperatures (AX40, AX60, and AX80). (**Left**): the intensity of autocorrelation functions versus delay time for the raw extracts with two different concentrations (points) and the Kohlrausch–Williams–Watts (KWW) function (Equation (1)) (lines). (**Right**): Reduced osmotic pressure $\frac{\pi }{c}$ as a function of extract concentration at different measurements temperatures (points) and the virial osmotic model (Equation (4)) (lines).

The complex molecular nature of the extracts was reflected in the DLS results. The autocorrelation functions $g^{2}\left(\tau \right)-1$ with the fit quality of the KWW model are shown in Figure 1 while the values of the diffusion coefficients ($D_{f}$ and $D_{s}$—Equation (1)) and the fractional contribution of fast phenomenon (*a*—Equation (1)) are given in Table 1.

For AX40 (extraction at 40 °C), the effect of extract concentration (0.2% and 1.0%) on the relaxation phenomena was most noticeable. The autocorrelation function for the 0.2% solution showed a tendency to disappear on the time scale of the experiment, and *a* value was of the order of 17%. In the case of 1.0% extract concentration, a non-decaying function of $g^{2}\left(\tau \right)-1$ was observed, and the fractional contribution *a* was of the order of 6%. The values of the diffusion coefficients (Table 1) representing fast and slow components were small, indicating the large size of the chains. The values given in the table should only be considered as an approximate measure of the phenomena—the conditions under which the measurements were carried out did not allow a precise determination of the hydrodynamic radius values (higher than 500 nm). For the extract obtained at 60 °C (AX60), we did not observe the effect of arabinoxylan concentration on the course of $g^{2}\left(\tau \right)-1$ (Figure 1) and there was no clear decay of the relaxation as a function of time. The most complex relaxation was observed for AX80: the shape of the autocorrelation function clearly indicated the lack of influence of the AX concentration and the complexity of the diffusion phenomenon, which is characterised by several contributors. The KWW model (Equation (1)) assumes that the diffusion was shaped by two main contributors; unfortunately, for AX60 and AX80, the results obtained did not fit well. The results for AX100 were not presented because they are not interpretable; $g^{2}\left(\tau \right)-1$ did not change in the experimental time, which could indicate a clear predominance of slow diffusion phenomena.

The colligative properties of AX solutions were studied at four different temperatures and the results are presented in the form of the concentration dependence of the reduced osmotic pressure $\frac{\pi }{c}$ in the graph (Figure 1) and the values of the average osmotic molecular mass ($M_{osm}$) as well as the second virial coefficient $A_{2}$ in Table 1. For AX40 solutions the reduced osmotic pressure decreases with increasing concentration. The exceptions were the results obtained at 30 °C, and the value of the second virial coefficient was positive (Table 1). Due to the small $\frac{\pi }{c}$ values in the lower concentration range, the average osmotic molecular mass ($M_{osm}$) extrapolated from the virial equation of state took a negative value. For measurements obtained at higher temperatures (34–40 °C), the $A_{2}$ values were negative, indicating that the AX chains had a higher affinity for each other than for the solvent. The values of the osmotic average molecular masses were more than 10 times higher than the weight average mass determined by GPC. The exception was the $M_{osm}$ determined from the results obtained at 38 °C. The reduced osmotic pressure reached its highest values at this temperature, varying from 0.8 mm H_2_O · dL · g^−1^ for the lowest AX concentration to 0.2 mm H_2_O · dL · g^−1^. Due to the largest change in $\frac{\pi }{c}$ with AX concentration, the $A_{2}$ value was the largest in absolute value. The measurements made for the AX60 fraction were of a similar nature, with values of reduced osmotic pressure being low and decreasing with increasing polysaccharide concentration. The exceptions were the measurements at 38 °C, where all $\frac{\pi }{c}$ values were negative and increased with AX concentration and, consequently, $A_{2}$ > 0. In this case the value of $M_{osm}$ was not estimated. At 30 °C, 34 °C, and 40 °C, the $A_{2}$ values were negative and increased in absolute value with temperature. The average osmotic molecular mass values decreased and were more than eight times higher than those determined by GPC. AX80 showed extremely different $\frac{\pi }{c}$ concentration relationships. For the lowest temperatures of 30 °C and 34 °C, the reduced osmotic pressure values were positive and decreased with increasing AX concentration. The $M_{osm}$ values were above 21,000 kDa and 6500 kDa (Table 1) and were only slightly higher than those determined by GPC. In contrast, at 38 °C and 40 °C the osmotic pressure became negative and increased with rising AX concentration. Due to $A_{2}$ > 0, the value of $M_{osm}$ was not estimated. It was not possible to measure the osmotic pressure of the AX100 fraction solutions due to the very high apparent viscosity of the solutions and the high instability of the signal.

**Figure 2:**
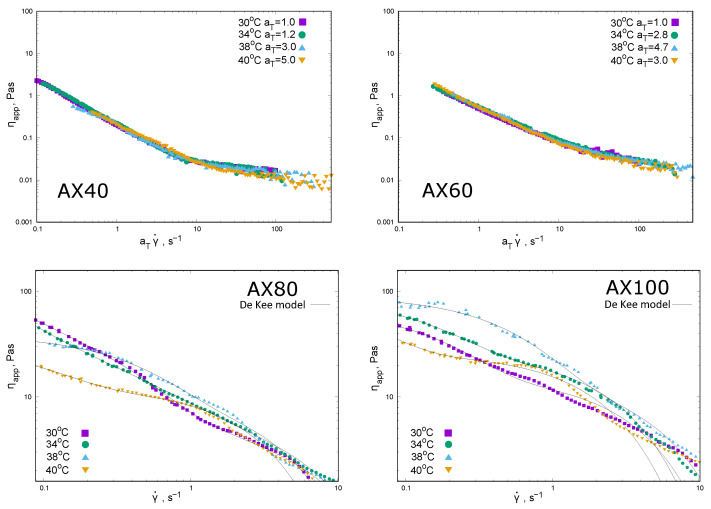
Rheological properties of raw extracts at the concentration of 1% obtained at different temperatures (AX40, AX60, AX80, and AX100). The dependence of apparent viscosity on the increasing shear rate (“up”) at different measurements temperatures (points). For AX40 and AX60, the dependencies of $\eta_{app}\left(\dot{\gamma }\right)$ were shifted with the help of temperature scaling coefficients $a_{T}$. Goodness of fit for De Kee model (Equation (6)) for AX80 and AX100.

**Figure 3:**
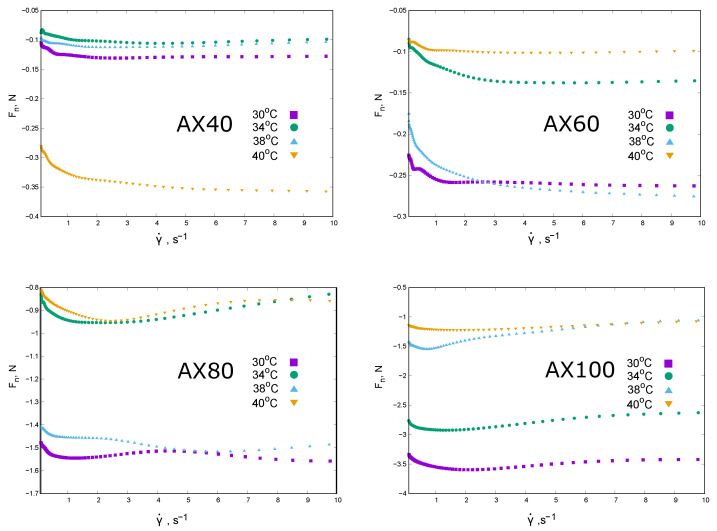
Rheological properties of raw extracts at the concentration of 1% obtained at different temperatures (AX40, AX60, AX80, and AX100). The dependence of normal force $F_{n}$ on the shear rate at different measurements temperatures. The experimental points represented values of $F_{n}$ obtained during “up” measurements under the conditions of increasing shear rate.

The rheological properties of AX extracts at a concentration of 1% are shown in the following graphs in terms of apparent viscosity $\eta_{app}$ (Figure 2) and normal force $F_{n}$ shear rate dependence (Figure 3).

The temperature scaling of the apparent viscosity as the shear rate dependence was applied to the results obtained for the AX40 and AX60 extracts (the values of the scaling coefficient $a_{T}$ are given in the graph description and in Table 1). The extracts AX40 and AX60 behaved as shear thinning systems (Figure 2); the discrepancy from the exponential model was small and visible in the range of higher shear rates. The rheological behavior of the fractions 1% AX80 and AX100 was more complex. The De Kee model was fitted to rheological data and two characteristic times were determined (Table 1). The evolution of the $\eta_{app}\left(\dot{\gamma }\right)$ relationship in the cases of both AX80 and AX100 indicated a number of contributions that shaped the rheological properties.

The values of normal force decreased non-linearly with the shear rate. It was also difficult to clearly determine the effect of the measurement temperature on the changes in $F_{n}\left(\dot{\gamma }\right)$. The normal force values were negative, which could be interpreted as the result of a collapse of the structure created by AX chains in solution or syneresis. The extracts of AX40 and AX60 showed the lowest absolute values of $F_{n}$. AX40 was least compressed at 34 °C, while under shear stress at 40 °C it was the most destroyed. A different behavior was observed in the case of AX60. The 1% extracts of AX80 and AX100 showed an even more complex dependence of $F_{n}\left(\dot{\gamma }\right)$: a minimum was observed, similar to AX100, except for the data obtained at 40 °C.

## 3. Discussion

Increasing the extraction temperature resulted in changes in the weight average molecular mass of the extracted fraction. Extraction at 25 °C [29] yielded an AX (AX25) fraction with the global maximum corresponding to $M_{w}$ = 220 kDa and the second peak $M_{w}$ = 1780 kDa. The use of higher extraction temperatures (40–100 °C) resulted in the extraction of AXs fractions characterised by much higher average molecular masses. Changes in the average molecular weight may be caused by variations in the arabinose and xylose content of the individual fractions. According to the available literature [10,21], the molar fraction of arabinose and xylose increases as the extraction temperature rises. The $M_{osm}$ values were significantly higher than the $M_{w}$ values, due to the high tendency of chains’ aggregation and high water absorption. The influence of water absorption on the osmotic pressure was described for polyelectrolyte solutions and hydrogels [32,33,34]. In the case of AX40, it reached the highest values, which, together with the negative $A_{2}$ values, could indicate a tendency of the chains to aggregate. The exceptions was the results obtained at 30 °C, where the $\frac{\pi }{c}$ values increased with the polysaccharide concentration. The $M_{osm}$ of AX60 were lower than those of AX40, while the $M_{w}$ of the AX60 was higher than that of AX40 (Table 1). According to the interpretation in [21], it was suggested that extensive interchain association is limited, with a higher A/X ratio. As the $A_{2}$ values were negative, it can be expected that the chains of the AX60 fraction aggregated. The phenomenon was least pronounced for AX80, which had the lowest osmotic average molecular masses. As the extraction temperature increased, the phenomenon associated with the occurrence of negative osmotic pressure values intensified. In terms of the operation of the membrane osmometer, this meant that the pressure on the membrane was created by the water flux from the solution side rather than the solvent side. This can be thought of as the result of water being “squeezed out” by the AX chains. At the same time, the $\frac{\pi }{c}$ values increased with AX concentration, which could mean that their affinity for the solvent increased. In general, the negative $A_{2}$ values confirming the limited solubility of AX in water were in line with the small values of the diffusion coefficients $D_{f}$ and $D_{s}$ indicating the presence of large objects in solution. This phenomenon was also observed by Yu et al. [30] and Ren et al. [21]. In order to understand the complex interactions between biopolymers and AX chains in aqueous solutions, a detailed explanation of these phenomena requires the combination of SEC-MALLS analysis with atomic force microscopy (AFM) and diffusing-wave spectroscopy (DWS) techniques.

Differences in the biopolymer–biopolymer interactions were also evident in the rheological behaviour. One percent AX40 and AX60 followed an exponential rheological model over the range of shear rates tested. AX80 and AX100 showed a complex behaviour that could be described by an De Kee model. The values of the time constants determined for these extracts indicated a combined mechanism for the formation of the rheological properties: times corresponding to viscous ($t_{1}$) and elastic ($t_{2}<<t_{1}$) contributions were present (Table 1). The rheological properties were mainly determined by viscous effects. It is possible that shorter time constants may have represented gel-like behaviour attributed to extracts obtained at higher temperatures [21]. Changes in the structure of the extracts’ behaviour were also evident in the values of the energy dissipated in the hysteresis loop experiment (Table 1). The predominance of viscous contributions is shown by the course of the (“up”) curve (above the down curve) and the lowest ΔE values—for AX40 and AX60. For AX80 and AX100, the ΔE values were much higher, mainly due to the complex course of flow curves reflecting the inhomogeneous structure of the fluid and the significant contribution of elastic properties.

The changes in the biopolymer–biopolymer interactions can also be explained on the basis of the average osmotic molecular mass values. The high $M_{osm}$ values obtained for AX40 and AX60—at least ten times the $M_{w}$—indicated not only a tendency for the chains to aggregate, but also the ability of these chains to associate water molecules-elasticity. Although this phenomenon decreased with increasing AX concentration (decreasing reduced osmotic pressure-$A_{2}<0$), in the light of the results presented in [21], it could be considered as a picture of a “weak gel” structure with a fibrous structure characterised by ordered and rigid chains cross-linked by weak connecting zones. Structurally, the extracts obtained were more susceptible to flow and showed a mainly viscous mechanism. This is probably the reason why the changes recorded in the values of the $F_{n}$ were the smallest in absolute value and did not change after a certain value of shear rate is exceeded (the amount of strain does not affect the rheological properties in the non-linear region).

The effect of temperature in the 30–40 °C range on the properties of 1% solutions is not clear. The first indication is a change in the sign of $A_{2}$. For all AX fractions there is a change in the nature of the dependence of $\frac{\pi }{c}$ on AX concentration in the temperature range studied. At 30 °C, AX40 solutions showed an osmotic pressure increasing with concentration, with $\frac{\pi }{c}$ values greater than zero. For AX60 and AX80, at selected temperatures, $\frac{\pi }{c}$ increases with concentration, but in a certain range of concentrations the osmotic pressure values are negative. This can be interpreted as a change in the nature of the interactions of the AX chains with water molecules. Perhaps as a result of the formation of internal hydrogen bridges, water molecules are “squeezed” out of the chain structure, resulting in a negative osmotic pressure value in the experiment. The intensification of this phenomenon with increasing extraction temperature, up to apparent syneresis in the case of AX100, would confirm the observations of Yu et al. [30] and Ren et al. [21] regarding the effect of extraction conditions on the structure of leached arabinoxylans. Analysis of the effect of measurement temperature on the rheological properties of 1% AX solutions is also inconclusive. For AX40 and AX60 an increase in temperature results in a decrease in apparent viscosity—as evidenced by the possibility of temperature scaling, solutions of the other fractions do not show such predictable changes. The ability to scale the apparent viscosity in the case of AX40 and AX60 may indicate an invariant mechanism governing the interactions between the chains. The only exception is $\eta_{app}$ at 40 °C, for which $a_{T}<a_{T=38^{\circ }C}$, which, in combination with the osmometry results ($A_{2}>0$) at 38 °C, may indicate a change in the structure of the AX60 solution. For AX80 and AX100 solutions there was no clear effect of temperature on the shear rate dependence of the $\eta_{app}$ and no temperature scaling could be performed. This behavior may indicate that the physical gel produced is characterised by a non-uniform structure.

## 4. Materials and Methods

### 4.1. Extract Preparation

Psyllium husk (*Plantago ovata* husk) (country of origin was India) was purchased in the local market (Radgeb Sp. z o.o., Wrocław, Poland). The composition in 100 g of the product (husk) declared by the supplier was the following: 1.93 g of proteins, 1.7 g of carbohydrates, 0.62 g of fat, and 85 g of dietary fibre. One gram of the husk was poured with 99 g of distilled water and the mixture was shaken for 2 h at four different temperatures: 40 °C (AX40), 60 °C (AX60), 80 °C (AX80), and 100 °C (AX100). After the specified time, the samples were centrifuged to separate the husk at 6000 rpm (centrifuge MPW-350R, MPW Med. Instruments, Warszawa, Poland) for 10 min. The protein content in the husk extract was determined using the Kjeldahl method according to the ISO standard. In all cases the protein content of the extract was less than 1.0%. The raw extracts obtained were used directly to determine the molecular mass.

Samples for osmometric measurements and dynamic light scattering (DLS) were prepared by appropriate dilution with distilled water of the initial extract to final concentrations of 0.2%, 0.3%, 0.4%, 0.5%, 0.6%, 0.8%, 0.9%, and 1.0%.

### 4.2. Determination of the Fractions Molecular Mass Distribution

The measurements of molecular masses distribution were performed by means of gel permeation chromatography (GPC) [35] at 25 °C. The chromatographic system consisted of two polymer-based columns of Ultrahydrogel-2000 and Ultrahydrogel-500 (Waters, Milford, MA, USA) connected in series and a refractometric detector RI (Knauer, Berlin, Germany). As an eluent, 0.1 mol/L $NaNO_{3}$ and 0.02% $NaN_{3}$ solution in water were applied. The flow rate was set to 0.6 mL/min, and the injection volume of the sample was 100 mL. The sample concentration was approximately 5 mg/mL. Calibration procedure according to the previously described method was performed using pullulan standards (Shodex, Tokyo, Japan).

### 4.3. Dynamic Light Scattering (DLS)

The 0.2% and 1.0% husk extracts were tested using dynamic light scattering at 25 °C on a Brookheven DLS/SLS system consisting of a BI-160 goniometer with digital autocorrelator BI-9000AT (Brookhaven, New York, NY, USA). As a source of light, a solid-state laser (JDSU, CDPS532M-050) with output power of 50 mW at λ = 532 nm was used. The light scattering angle chosen for measurements was 90°. Three repetitions were performed for all samples. The time average intensity correlation function $\left[g^{2}\left(\tau \right)-1\right]$ was obtained with the acquisition time of 300 s for each run with the help of Brookhaven Instruments Dynamic Light Scattering Software version 5.9. Mathematical modelling of hydrodynamic properties was carried out with the use of Kohlrausch–Williams–Watts (KWW) [36] stretched exponential function:

(1) $${\left[g^{2}\left(\tau \right)-1\right]}_{KWW}\approx {\left\{a\cdot exp\left(\frac{-\tau }{\tau_{f}}\right)+\left(1-a\right)\cdot exp{\left[-\left(\frac{\tau }{\tau_{s}}\right)\right]}^{\beta }\right\}}^{2}$$

where $g^{2}\left(\tau \right)-1$ is the intensity autocorrelation function; $\tau_{f}$, $\tau_{s}$ are relaxation times of the fast (f) and slow (s) components, respectively; β is the exponent of the stretched exponential; τ is the delay time; and *a* and (1−a) represent the fractional contribution of the two processes. Estimation of parameters (Equation (1)) was done according to the Levenberg–Marquardt algorithm using the least squares method:

(2) $$\chi_{KWW}^{2}=\Sigma {\left\{\left[g^{2}\left(\tau \right)-1\right]-{\left[g^{2}\left(\tau \right)-1\right]}_{KWW}\right\}}^{2}⟶min$$

The diffusion coefficients for slow $D_{s}$ and fast $D_{f}$ components were calculated according to

(3) $$D_{k}=\frac{1}{\tau_{k}\cdot q^{2}},k=s,f$$

where $q=\frac{4\pi n}{\lambda }\cdot sin\left(\frac{\theta }{2}\right)$ is the value of the magnitude of the scattering wave vector.

### 4.4. Osmotic Pressure Measurements

Osmotic pressure measurements of husk extracts were performed with an OSMOMAT 090 membrane osmometer (Gonotec, Berlin, Germany) using a double-layer cellulose membrane with a cut-off value of 20,000 Da. Measurements were carried out at four selected temperatures: 30 °C, 34 °C, 38 °C, and 40 °C, to the nearest 0.1 K. Four repetitions were performed for all extracts (concentrations and temperatures). Such an extensive research program enabled the estimation of the parameters of the osmotic equation of state:

(4) $$\frac{\pi }{c}=\frac{RT}{M_{osm}}\cdot \left[1+A_{2}\left(T\right)\cdot c+A_{3}\left(T\right)\cdot c^{2}+A_{4}\left(T\right)\cdot c^{3}\right]$$

where π is the osmotic pressure, mm H_2_O; *c* is the concentration of the polysaccharide, g/100 mL; *R* is the gas constant, *T* is temperature, $M_{osm}$, kg · mol^−1^ is the average osmotic molecular mass, and $A_{2}\left(T\right)$, $A_{3}\left(T\right)$, cm^3^· mol^−1^· g^−2^ are the second and third osmotic virial coefficients. Estimation of osmotic state equations parameters were carried out using the nonlinear algorithm of Marquardt–Levenberg. The target function was formulated as

(5) $$\chi_{L-M}^{2}=\Sigma {\left[\frac{\pi^{\sigma }}{c}-\frac{\hat{\pi }}{c}\right]}^{2}⟶min$$

where $\frac{\pi^{\sigma }}{c}$ are values of the experimental reduced osmotic pressure and $\frac{\hat{\pi }}{c}$ were calculated from Equation (4). The minimisation procedure estimated the values of the average osmotic molecular mass ($M_{osm}$) and values of the osmotic virial coefficient based on experimental data obtained at each one temperature for all extracts.

### 4.5. Rheological Properties

The rheological characteristic of the husk extracts at the concentration of 1% was determined using the rotational rheometer RS6000 (ThermoFisher, Karlsruhe, Germany) with the cone-plate geometry sensor ($d_{in}$ = 60 mm, 1°). The hysteresis tests at 30 °C, 34 °C, 38 °C, and 40 °C were performed, with an increasing (“up”) and decreasing (“down”) shear rate of 1 s^−1^ to 100 s^−1^. Time of each stage was of 300 s. During the experiment, data on changes in the normal force value ($F_{n}$) were also collected. Three repetitions were performed for all extracts tested. The amount of the energy dissipated (ΔE,J) was calculated using the value of area between flow curves obtained during increasing and decreasing shear rate (Pa · s^−1^), the time of the experiment (Δt,s), and the volume of sample ($V=2.9688\cdot 10^{-7}$ m^3^) according to the equation ΔE=P·Δt·V.

#### Mathematical Analysis of Rheological Results

The rheological properties were described with the help of the De Kee model [37]:

(6) $$\eta \left(\dot{\gamma }\right)=\tau_{0}{\dot{\gamma }}^{-1}+\Sigma_{p=1}^{\infty }\eta_{p}\exp\left(-t_{p}\dot{\gamma }\right)$$

The estimated parameters were yield stress $\tau_{0}$ (Pa), the exponents $t_{p}$ with time dimension, and coefficients $\eta_{p}$p=1,2 with dimension of viscosity. In the case of estimating a larger number of pairs ($t_{p},\eta_{p}$), the results can be presented in the form of a discrete distribution of time constants $\eta_{p}\left(t_{p}\right)$. Coefficient values of $\eta_{p}$ described the intensity of the time constants $t_{p}$. Estimation of parameters (Equation (6)) was according to the Marquardt–Levenberg method, which was applied as the minimisation algorithm using the least squares method. The target function is defined as follows:

(7) $$\chi^{2}=\Sigma {\left[\eta^{\sigma }-\hat{\eta }\right]}^{2}⟶min$$

where $\eta^{\sigma }$ are experimental values of apparent viscosity; $\hat{\eta }$ were calculated from Equation (6).

### 4.6. Data Analysis and Statistics

The method of data analysis was based on the Marquardt–Levenberg non-linear least-square iteration procedure. The parameters standard errors were estimated from the variance–covariance matrix after the final iteration. Calculations were carried out using software prepared in Python 3.11.4.

## 5. Conclusions

The increasing temperature of extraction caused rising values of weight average molecular mass. The colligative and hydrodynamic properties of the AX fractions extracted at four different temperatures varied, most likely due to differences in the biopolymer–biopolymer and biopolymer–water interactions of the extracted arabinoxylans. It was supported by the DLS and rheological results. Only for AX40 were differences in the autocorrelation functions found at lower concentration. For the other fractions, the autocorrelation functions determined were virtually the same regardless of concentration and did not disappear during the experiment. The interactions created by the chains in solution were characterised by very long relaxation times and a small contribution of the fast mode in shaping the relaxation phenomenon. The complex nature of biopolymer–water interactions was also manifested in the effects of concentration and temperature on changes in osmotic pressure of extracts. The dominance of biopolymer–biopolymer interactions was evident in the negative values of the second virial coefficient, which is indicative of the tendency of biopolymer chains to aggregate. The rheological properties of the extracts changed from being described by a power-law model at 40 °C and 60 °C to being described by the full non-linear De Kee model at 80 °C and 100 °C.

## Figures and Tables

**Table 1:** Molecular parameters (average molecular masses $M_{w}$, $M_{n}$, and dispersity $P_{d}$), hydrodynamic (*a*—fractional contribution (Equation (1), $D_{f}$, $D_{s}$—diffusion coefficients (Equation (3)) and osmotic ($A_{2}$—second virial coefficient, $M_{osm}$—osmotic average molecular mass (Equation (4))) characteristics of extracted at different temperatures ($T_{ex}$) mucilage fractions (AX40, AX60, AX80, and AX100) and rheological parameters of the De Kee model ($t_{1}$, $t_{2}$—time constants, $\tau_{0}$—yield stress (Equation (6))), scaling coefficients ($a_{T}$), and the amount of energy dissipated ΔE. The goodness of fit for Equations (1)–(6) is given in Appendix A (Table A1).

	$T_{\mathrm{ex}}$	$M_{w}$	$M_{n}$	$P_{d}$	a	$D_{f}\cdot 10^{-9}$	$D_{s}\cdot 10^{-12}$	$T_{\mathrm{osm}}$	$A_{2}\cdot 10^{-9}$	$M_{\mathrm{osm}}$	ΔE	$t_{1}$	$t_{2}$	$\tau_{0}$	$a_{T}$
	°C	kDa	kDa			cm^2^· s^−1^	cm^2^· s^−1^	°C	cm^3^· mol^−1^ · g^−2^	kDa	mJ	s	s	Pa	
AX40	40	2190	1423	2.05	0.06	1.04	5.34	30	32	-	2.44				1.0
								34	−26	105,000	2.20				1.2
								38	−124	19,400	0.91				3.0
								40	−18	80,700	0.43				5.0
AX60	60	3160	1513	2.09	0.14	0.90	0.21	30	−116	27,600	3.16				1.0
								34	−58	69,200	1.52				2.8
								38	9.0	-	1.80				4.7
								40	−126	27,280	1.40				3.0
AX80	80	3320	1338	2.48	0.31	4.78	0.32	30	−143	21,000	28.0	3.1	0.2	2.8	
								34	−580	6500	132	1.6	0.2	3.2	
								38	20	-	48.0	2.3	0.3	-	
								40	5	-	14.0	10.7	0.4	-	
AX100	100	3320	1656	2.00	-	-	-	30	-	-	8.40	5.9	0.3	-	
								34	-	-	44.0	6.6	0.4	-	
								38	-	-	320	1.9	0.2	-	
								40	-	-	48.0	21.0	0.5	-	

## Data Availability

Data are contained within the article.

## Appendix A

### Appendix A.1

**Table A1:** The goodness of fit for the Kohlrausch–Williams–Watts stretched exponential function (Equation (1)), osmotic equation of state (Equation (4)), and De Kee rheological model (Equation (6)).

	Equation (1)				Equation (4)				Equation (6)
Sample	a	$D_{f}$	$D_{s}$	$\chi_{\mathrm{KWW}}^{2}$	$T_{\mathrm{osm}}$	$A_{2}$	$M_{\mathrm{osm}}$	$\chi_{M-L}^{2}$	$\chi^{2}$
AX40	±1.6%	±3.4%	±2.8%	0.00017	30	±18%	±9%	0.0040	-
					34	±14%	±9%	0.0010	-
					38	±13%	±9%	0.0000	-
					40	±16%	±9%	0.0004	-
AX60	±1.4%	±4.2%	±5.0%	0.00132	30	±18%	±7%	0.0006	-
					34	±16%	±9%	0.0011	-
					38	±16%	-	0.0013	-
					40	±14%	±9%	0.0005	-
AX80	±1.1%	±9.7%	±5.4%	0.00517	30	±18%	±12%	0.0013	0.0022
					34	±14%	±13%	0.0155	0.0021
					38	±13%	-	0.0012	0.0031
					40	±19%	-	0.0023	0.0143
AX100	-	-	-	-	30	-	-	-	0.2013
	-	-	-	-	34	-	-	-	0.2415
	-	-	-	-	38	-	-	-	0.1942
	-	-	-	-	40	-	-	-	0.3941

## References

[1] Fradinho P., Nunes M.C., Raymundo A. (2015). Developing consumer acceptable biscuits enriched with Psyllium fibre. J. Food Sci. Technol..

[2] Franco E.A.N., Sanches-Silva A., Ribeiro-Santos R., de Melo N.R. (2020). Psyllium (*Plantago ovata* Forsk): From evidence of health benefits to its food application. Trends Food Sci. Technol..

[3] Franco E.A.N., Chávez D.W.H., de Lima A.B., do Socorro Rocha Bastos M., de Melo N.R. (2023). Psyllium (*Plantago ovata* Forsk) in frozen banana pulp: Influence on rheological, nutritional and sensory characteristics. Food Sci. Technol Int..

[4] Pitkänen L., Virkki L., Tenkanen M., Tuomainen P. (2009). Comprehensive Multidetector HPSEC Study on Solution Properties of Cereal Arabinoxylans in Aqueous and DMSO Solutions. Biomacromolecules.

[5] Pérez-Flores J.G., García-Curiel L., Pérez-Escalante E., Contreras-López E., Olloqui E.J. (2024). Arabinoxylans matrixes as a potential material for drug delivery systems development—A bibliometric analysis and literature review. Heliyon.

[6] Kumar D., Pandey J.S., Kumar P.S., Raj V.V. (2017). Psyllium Mucilage and Its Use in Pharmaceutical Field: An Overview. Curr. Synth. Syst. Biol..

[7] Mendez-Encinas M.A., Carvajal-Millan E., Rascon-Chu A., Astiazaran-Garcia H.F., Valencia-Rivera D.E. (2018). Ferulated Arabinoxylans and Their Gels: Functional Properties and Potential Application as Antioxidant and Anticancer Agent. Oxidative Med. Cell. Longev..

[8] Fadel A., Plunkett A., Li W., Tessu Gyamfi V.E., Nyaranga R.R., Fadel F., Dakak S., Ranneh Y., Salmon Y., Ashworth J.J. (2018). Modulation of innate and adaptive immune responses by arabinoxylans. J. Food Biochem..

[9] Fischer M.H., Yu N., Gray G.R., Ralph J., Anderson L., Marlett J.A. (2004). The gel-forming polysaccharide of psyllium husk (*Plantago ovata* Forsk). Carbohydr. Res..

[10] Yu L., Yakubov G.E., Zeng W., Xing X., Stenson J., Bulone V., Stokes J.R. (2017). Multi-layer mucilage of *Plantago ovata* seeds: Rheological differences arise from variations in arabinoxylan side chains. Carbohydr. Polym..

[11] Gharibzahedi S.M.T., Smith B., Guo Y. (2019). Pectin extraction from common fig skin by different methods: The physicochemical, rheological, functional, and structural evaluations. Int. J. Biol. Macromol..

[12] Ji X., Hou C., Yan Y., Shi M., Liu Y. (2020). Comparison of structural characterization and antioxidant activity of polysaccharides from jujube (*Ziziphus jujuba* Mill.) fruit. Int. J. Biol. Macromol..

[13] Ying Z., Han X., Li J. (2011). Ultrasound-assisted extraction of polysaccharides from mulberry leaves. Food Chem..

[14] Hashemifesharaki R., Xanthakis E., Altintas Z., Guo Y., Gharibzahedi S.M.T. (2020). Microwave-assisted extraction of polysaccharides from the marshmallow roots: Optimization, purification, structure, and bioactivity. Carbohydr. Polym..

[15] Li C., Mao X., Xu B. (2013). Pulsed electric field extraction enhanced anti-coagulant effect of fungal polysaccharide from Jew’s ear (*Auricularia auricula*). Phytochem. Anal..

[16] Liu J., Li Y., Liu W., Qi Q., Hu X., Li S., Lei J., Rong L. (2019). Extraction of Polysaccharide from Dendrobium nobile Lindl. By Subcritical Water Extraction. ACS Omega.

[17] Maes C., Delcour J.A. (2002). Structural characterisation of water-extractable and water-unextractable arabinoxylans in wheat bran. J. Cereal Sci..

[18] Ayala-Soto F.E., Serna-Saldívar S.O., Welti-Chanes J. (2016). Effect of processing time, temperature and alkali concentration on yield extraction, structure and gelling properties of corn fiber arabinoxylans. Food Hydrocoll..

[19] Rostami H., Gharibzahedi S.M.T. (2017). Cellulase-assisted extraction of polysaccharides from *Malva sylvestris*: Process optimization and potential functionalities. Int. J. Biol. Macromol..

[20] Craeyveld V.V., Delcour J.A., Courtin C.M. (2009). Extractability and chemical and enzymic degradation of psyllium (*Plantago ovata* Forsk) seed husk arabinoxylans. Food Chem..

[21] Ren Y., Yakubov G.E., Linter B.R., MacNaughtan W., Foster T.J. (2020). Temperature fractionation, physicochemical and rheological analysis of psyllium seed husk heteroxylan. Food Hydrocoll..

[22] Guo Q., Cui S.W., Wang Q., Young J.C. (2008). Fractionation and physicochemical characterization of psyllium gum. Carbohydr. Polym..

[23] Guo Q., Cui S.W., Wang Q., Goff H.D., Smith A. (2009). Microstructure and rheological properties of psyllium polysaccharide gel. Food Hydrocoll..

[24] Addoun N., Boual Z., Delattre C., Ursu A.V., Desbrières J., Le Cerf D., Gardarin C., Hentati F., El-Hadj M.D.O., Michaud P. (2020). Structural features and rheological behavior of a water-soluble polysaccharide extracted from the seeds of Plantago ciliata Desf. Int. J. Biol. Macromol..

[25] Adams E.L., Kroon P.A., Williamson G., Morris V.J. (2003). Characterisation of heterogeneous arabinoxylans by direct imaging of individual molecules by atomic force microscopy. Carbohydr. Res..

[26] Qaisrani T.B., Qaisrani M.M., Qaisrani T.M. (2016). Arabinoxylans from psyllium husk: A review. J. Environ. Agric. Sci..

[27] Patel M.K., Tanna B., Mishra A., Jha B. (2018). Physicochemical characterization, antioxidant and anti-proliferative activities of a polysaccharide extracted from psyllium (*P. ovata*) leaves. Int. J. Biol. Macromol..

[28] Farahnaky A., Askari H., Majzoobi M., Mesbahi G. (2010). The impact of concentration, temperature and pH on dynamic rheology of psyllium gels. J. Food Eng..

[29] Kaczmarczyk K., Kruk J., Ptaszek P., Ptaszek A. (2023). Plantago Ovata Husk: An Investigation of Raw Aqueous Extracts. Osmotic, Hydrodynamic and Complex Rheological Characterisation. Molecules.

[30] Yu L., Yakubov G.E., Martínez-Sanz M., Gilbert E.P., Stokes J.R. (2018). Rheological and structural properties of complex arabinoxylans from *Plantago ovata* seed mucilage under non-gelled conditions. Carbohydr. Polym..

[31] Shelat K.J., Vilaplana F., Nicholson T.M., Wong K.H., Gidley M.J., Gilbert R.G. (2010). Diffusion and viscosity in arabinoxylan solutions: Implications for nutrition. Carbohydr. Polym..

[32] Rubinstein M., Colby R.H., Dobrynin A.V., Joanny J.F. (1996). Elastic modulus and equilibrium swelling of polyelectrolyte gels. Macromolecules.

[33] Bay L., Jacobsen T., Skaarup S., West K. (2001). Mechanism of actuation in conducting polymers: Osmotic expansion. J. Phys. Chem. B.

[34] Bhattacharyya A., O’Bryan C., Ni Y., Morley C.D., Taylor C.R., Angelini T.E. (2020). Hydrogel compression and polymer osmotic pressure. Biotribology.

[35] Lukasiewicz M., Bednarz S., Ptaszek A. (2011). Environmental friendly polysaccharide modification – microwave-assisted oxidation of starch. Starch/Stärke.

[36] Shibayama M., Tsujimoto M., Ikkai F. (2000). Static Inhomogeneities in Physical Gels: Comparison of Temperature-Induced and Concentration-Induced Sol-Gel Transition. Macromolecules.

[37] Kee D.D., Turcotte G. (1980). Viscosity of biomaterials. Chem. Eng. Commun..

